# A descriptive analysis of skin-only closure and Bogota bag techniques for achieving complete fascial closure in damage control abdominal surgery

**DOI:** 10.1186/s12893-024-02484-2

**Published:** 2024-06-20

**Authors:** Muhammad Jawad Zahid, Musarrat Hussain, Dileep Kumar, Muhammad Hamza, Syed Amir Zeb Jan, Haadia Safdar, Jithin Kochupurackal Ajith, Ira Prakarsh, Wireko Andrew Awuah

**Affiliations:** 1 Hayatabad Medical Complex, Peshawar, Pakistan; 2 Sir Syed College of Medical Sciences for Girls, Karachi, Pakistan; 3 Advanced International Hospital, Islamabad, Pakistan; 4 Medway NHS Foundation Trust, Kent, England; 5 University of Dundee, Dundee, Scotland; 6 Sumy State University, Sumy, Ukraine

**Keywords:** Temporary abdominal closure, Skin-only closure, Bogota bag closure, Primary fascial closure, Peritonitis, Abdominal trauma

## Abstract

**Background:**

Temporary abdominal closure (TAC) techniques are essential in managing open abdomen cases, particularly in damage control surgery. Skin-only closure (SC) and Bogota bag closure (BBC) are commonly used methods for TAC, but their comparative effectiveness in achieving primary fascial closure (PFC) remains unclear. The objective of this study was to evaluate the rates of PFC between patients undergoing SC and BBC techniques for TAC in peritonitis or abdominal trauma cases at a tertiary care hospital.

**Methods:**

A retrospective cross-sectional study was conducted at the Surgical A Unit of Hayatabad Medical Complex, Peshawar, from January 2022 to July 2023. Approval was obtained from the institutional review board, and patient consent was secured for data use. Patients undergoing temporary abdominal closure using either skin-only or Bogota bag techniques were included. Exclusions comprised patients younger than 15 or older than 75 years, those with multiple abdominal wall incisions, and those with prior abdominal surgeries. Data analysis utilized SPSS version 25. The study aimed to assess outcomes following damage control surgery, focusing on primary fascial closure rates and associated factors. Closure techniques (skin-only and Bogota bag) were chosen based on institutional protocols and clinical context. Indications for damage control surgery (DCS) included traumatic and non-traumatic emergencies. Intra-abdominal pressure (IAP) was measured using standardized methods. Patients were divided into SC and BBC groups for comparison. Criteria for reoperation and primary fascial closure were established, with timing and technique determined based on clinical assessment and multidisciplinary team collaboration. The decision to leave patients open during the index operation followed damage control surgery principles.

**Results:**

A total of 193 patients were included in this study, with 59.0% undergoing skin-only closure (SC) and 41.0% receiving Bogota bag closure (BBC). Patients exhibited similar demographic characteristics across cohorts, with a majority being male (73.1%) and experiencing acute abdomen of non-traumatic origin (58.0%). Among the reasons for leaving the abdomen open, severe intra-abdominal sepsis affected 51.3% of patients, while 42.0% experienced hemodynamic instability. Patients who received SC had significantly higher rates of primary fascial closure (PFC) compared to BBC (85.1% vs. 65.8%, *p* = 0.04), with lower rates of fascial dehiscence (1.7% vs. 7.6%, *p* = 0.052) and wound infections (*p* = 0.010). Multivariate regression analysis showed SC was associated with a higher likelihood of achieving PFC compared to BBC (adjusted OR = 1.7, 95% CI: 1.3–3.8, *p* < 0.05).

**Conclusion:**

In patients with peritonitis or abdominal trauma, SC demonstrated higher rates of PFC compared to BBC for TAC in our study population. However, further studies are warranted to validate these results and explore the long-term outcomes associated with different TAC techniques.

## Introduction

Temporary abdominal closure (TAC) is a technique to manage an open abdomen for planned second-look laparotomies [1]. Its use became more common with the establishment of the concept of damage control surgery. Temporary abdominal closure keeps the abdominal viscera secure, while the paired rectus muscles are not approximated [2]. It mitigates fluid losses and infectious complications and prevents the development of abdominal compartment syndrome and enterocutaneous fistulas [3]. The four categories of temporary abdominal closure techniques are skin-only closure, Bogota-bag closure, patch closures (mesh, Wittmann patch, or zipper), and vacuum-assisted closure, each with its own advantages and disadvantages [4]. 

The two (go-to) techniques for temporary abdominal closure in northern Pakistan are the Bogota bag and skin-only closure. Both are quick and cheap, and they are mostly used interchangeably in most hospitals. Skin-only closure has lesser fluid losses but has become less popular due to recent evidence of higher rates of intraabdominal hypertension, infections, and enterocutaneous fistulas [2–5]. While the Bogota bag closure allows for farther retraction of the recti and, depending on the material, may allow some visualization of abdominal organs and detection of gross blood or fluid losses [1]. Some surgeons prefer the Bogota bag over skin-only closure because of the dreaded complication of failure to achieve primary fascial closure. Though some data suggests that skin-only closure results in higher rates of primary fascial closure, other studies have also reported equal outcomes for both techniques [6, 7]. 

This study intends to address a significant research gap by describing the rates of complete fascial approximation between two techniques: skin-only closure and Bogota bag closure, in a cohort of patients undergoing abdominal surgery. To the best of our knowledge, this specific comparison has not been investigated in our patient population. Therefore, our objective is to contribute valuable insights into determining the most effective technique for abdominal closure.

## Methodology

This retrospective cross-sectional study analyzed medical records from the Surgical A Unit of the Hayatabad Medical Complex, Peshawar, covering January 2022 to July 2023. Institutional review board approval was obtained (HMC-QAD-F-00-1649), and patients provided consent through the institutional form for data utilization in research. The included patients underwent temporary abdominal closure (TAC) using either skin-only or Bogota bag techniques. Surgical procedures were performed by a consistent team to ensure uniform expertise. Exclusions comprised patients aged < 15 or > 75 years with multiple abdominal incisions and prior abdominal surgeries. Surviving patients formed the study cohort. Data collection spanned hospitalization duration without considering follow-up settings, was gathered via electronic forms, and analyzed using SPSS version 25.

To provide context for our study, it is essential to understand the setting in which it was conducted. At Hayatabad Medical Complex, located in a resource-constrained environment, the management of abdominal septicemia and severe abdominal trauma presents a considerable burden. With an annual caseload of 400–500 laparotomies, a quarter to a third of our patients require open abdomen treatment. This high volume reflects the prevalence of these conditions in our region and the challenges faced by our surgical team.

## Rationale for technique selection

The choice of these methods was based on institutional protocols and the specific clinical context encountered in our setting. The choice between SC and BBC often depends on the surgeon’s assessment of the patient’s condition, the extent of abdominal contamination, and the expected duration until definitive closure can be achieved. Factors such as limited resources, including financial constraints and the availability of supplies, as well as the complexity of abdominal pathologies, influenced our decision. While we acknowledge that other closure techniques may be favored by many surgeons, we opted for SC and BBC closures due to their demonstrated effectiveness, particularly in resource-constrained environments or when managing complex abdominal cases.

## Indications for damage control surgery (DCS)

Damage control surgery (DCS) was primarily indicated for traumatic injuries, including penetrating or blunt abdominal trauma resulting from gunshot or stab wounds, motor vehicle accidents, or falls, leading to solid organ or hollow viscus damage. Additionally, non-traumatic intra-abdominal emergencies, such as acute mesenteric ischemia, ischemic bowel, bowel obstruction, or perforated viscus like peptic ulcer perforations, necessitated prompt surgical intervention.

### Decision for open abdomen management

The decision to employ damage control surgery and leave patients with an open abdomen during the index operation was based on intraoperative considerations. These included the presence of severe intra-abdominal sepsis, hemodynamic instability, compromised tissue perfusion, and extensive tissue loss. Adhering to the principles of damage control surgery, this approach aimed to achieve source control and hemodynamic stabilization in critically ill patients while minimizing the risk of complications associated with immediate fascial closure, such as abdominal compartment syndrome.

### Measurement of intra-abdominal pressure (IAP)

IAP was measured using standardized intravesical pressure methods. A urinary catheter was inserted to measure bladder pressure reflecting IAP, ensuring monitoring of pressure changes [8]. Clinical assessment complements pressure measurements for a comprehensive evaluation.

### Study population and groups

The study population was divided into two groups: the skin-only closure group (SC) and the Bogota bag closure group (BBC). A comparison was made between the two groups regarding demographics and other independent and dependent clinical variables. The chi-square test was used for nominal variables, and either the T-test or the Mann-Whitney U test was employed for interval variables, depending on the distribution of the data. A p-value of less than 0.05 was considered statistically significant.

### Definition of primary fascial closure

Primary fascial closure is defined as the initial approximation of the fascial edges through suture repair without the utilization of adjunctive techniques such as mesh reinforcement or component separation. Additionally, successful primary fascial closure is characterized by the absence of fascial dehiscence during the patient’s initial hospitalization period [7, 9, 10]. 

### Criteria and timing for primary fascial closure

Decisions for reoperation and primary fascial closure were based on a combination of clinical assessments and the patient’s physiological status. The criteria for reoperation included ongoing infection, hemodynamic instability, and signs of abdominal compartment syndrome. Specific criteria for attempting primary fascial closure involved the resolution of physiological derangement, control of sepsis, reduction of intra-abdominal pressure, absence of ongoing bowel ischemia, improvement in tissue perfusion, reduction in bowel edema, and optimal wound conditions. These criteria align with the principles of damage control surgery and aim to ensure patient safety and successful closure.

The timing for primary fascial closure was typically within 3–4 days post-surgery, depending on the patient’s condition. The suitability for primary fascial closure was assessed based on the absence of ongoing contamination, resolution of abdominal edema, stabilization of intra-abdominal pressure, and the absence of ongoing hemorrhage. The decision for primary fascial closure was made in collaboration with a multidisciplinary team involving surgeons, intensivists, and wound care specialists. In cases where primary fascial closure was not achieved, options included temporary mesh closure or planned ventral hernia repair.

### Closure technique employed

The technique employed for primary fascial closure involved the use of continuous or interrupted sutures to approximate the fascial edges in a tension-free manner. The specific suture material and technique were selected based on surgeon preference and the patient’s individual characteristics, with the primary goal of achieving secure fascial approximation while minimizing the risk of postoperative complications.

## Results

A total of 193 patients were eligible for inclusion in the study, and they exhibited similar demographic characteristics across the cohorts. Among the included patients, 59.0% underwent treatment with the skin-only closure (SC) technique, while the remaining 41.0% received the Bogota bag closure (BBC) technique during the study period. The majority of the patients were male (73.1%), and a higher proportion experienced acute abdomen of non-traumatic origin (58.0%). The two groups had a notable overlap regarding the reasons for leaving the abdomen open. Among the primary reasons cited were severe intra-abdominal sepsis, which affected 51.3% of patients, and hemodynamic instability, noted in 42.0% of cases. Additionally, a smaller subset of patients, comprising 6.7%, required open abdomen management due to extensive tissue loss. This overlap underscores the complexity and severity of the clinical presentations encountered in our cohort, necessitating tailored approaches to optimize patient outcomes (Table 1).

**Table 1:** Baseline Demographics and preoperative variables of the included cohort

Demographic and Preoperative Variables				
Variables	Overall (*n* = 193)	SC (*n* = 114)	BBC (*n* = 79)	*P*-value
**Gender**				
Males (%)	141 (73.1)	85(74.5)	56(71)	0.571
Females (%)	52(26.9)	29(25.4)	23(29.1)	
**Age**				
Mean Age+/-SD	52.54 ± 13.84	51.04 ± 14.58	54.05 ± 13.10	0.144
Age > 40 (%)	145(75)	82(71.9)	63(79.7)	0.217
Age < 40 (%)	48(25)	32(28.1)	16(20.3)	
**Surgical Indication**				
Surgery for acute abdomen of non-traumatic origin (%)	112(58)	68(59.6)	44(55.7)	0.584
Acute Mesenteric Ischemia	28	17	11	
Acute Abdomen due to Ischemic Bowel or Bowel Obstruction	69	44	25	
Perforated Viscus like Peptic Ulcer Perforations	15	7	8	
Surgery for Trauma (%)	81(42)	46(40.4)	35(44.3)	
**Reasons for leaving abdomen open (n%)**				
Severe Intra-Abdominal Sepsis	99 (51.2)	61 (53.6)	38 (48.1)	-
Hemodynamic Instability	81 (41.9)	49 (43.0)	32 (40.5)	-
Extensive Tissue Loss*	13 (6.7)	4 (3.5)	9(11.4)	-
**Comorbidities**				
Diabetes (%)	38(19.7)	18(15.7)	20(25.3)	0.102
Tobacco Use (%)	86(44.5)	52(45.6)	34(43.0)	0.723
Hypertension (%)	54 (28.0)	28 (24.6)	26 (32.9)	0.217
CAD (%)	29 (15.0)	14 (12.3)	15 (19.0)	0.356
**Preoperative Shock (%)**	95(49.2)	40(35.1)	55(69.6)	0.312
**Preoperative Anemia (%)**	79(41)	33(29.9)	46(58.2)	0.081

SC: Skin-closure only; BBC: Bogota bag closure

*Extensive tissue loss refers to cases where debridement and/or abdominal wall resection were performed, as part of the indication for temporary abdominal closure (TAC).

There was a statistically significant difference (*P* = 0.015) between the SC and BBC groups for the patients undergoing more than two surgeries (42.3% vs. 60.8%). Further, patients in the SC group required fewer blood transfusions (0.81 ± 0.863) compared to the Bogota Bag Closure (BBG) group (1.25 ± 0.992) (*p* = 0.001). Although not statistically significant, the SC group required more vasopressors (32.4% vs. 27.8%) and less ventilator support (42.1% vs. 55.7%). Furthermore, a higher proportion of patients in the BBC group required external tension sutures compared to the SC group (39.2% vs. 23.6%, *P* = 0.017) (Table 2).

**Table 2:** Intraoperative and Surgical Variables

Intraoperative and surgical variables				
Variables	Overall (*n* = 193)	SC (*n* = 114)	BBC (*n* = 79)	*P*-value
Number of Surgeries: <2 (n,%)	96 (49.7)	65(57.0)	31(39.2)	0.015
Number of Surgeries > 2 (n,%)	97(50.3)	49(42.3)	48 (60.8)	
Number of Blood Transfusions (Mean ± SD)	1.03 ± 0.92	0.81 ± 0.863	1.25 ± 0.992	0.001
Need for Vasopressors (n,%)	59(30.5)	37(32.4)	22(27.8)	0.494
Need for Ventilator Support (n,%)	92(47.6)	48(42.1)	44(55.7)	0.063
Days to Closure (Mean ± SD)	3.36 ± 1.455	3.34 ± 1.456	3.39 ± 1.454	0.814
Positive Blood Cultures (n,%)	70(36.3)	38(54.2)	32(45.8)	0.308
Use of External Tension Sutures (n,%)	58(30)	27(23.6)	31(39.2)	0.017
ICU Stay (Mean ± SD)	7.3 ± 3.62	6.9 ± 3.81	7.5 +/-3.23	0.151
Hospital Stay (Mean ± SD)	12.1 ± 4.91	11.31 ± 5.25	13.7+/-4.11	0.310

SC: Skin-closure only; BBC: Bogota bag closure; ICU: Intensive Care Unit

In terms of primary fascial closure rates, the skin-closure only (SC) group exhibited a significantly higher rate (85.1%) compared to the Bogota bag closure (BBC) group (65.8%) (*p* = 0.04). Additionally, wound infections were significantly more frequent in the BBC group (30.3%) compared to the SC group (15%) (*p* = 0.010) (Table 3).

**Table 3:** Primary and Secondary Outcomes

Primary and Secondary Outcomes			
Outcomes	SC (*n* = 114)	BBC (*n* = 79)	*P*-value
Primary Fascial Closure	97(85.1)	52(65.8)	0.04
Intra-abdominal Abscess Formation	6(5.3)	9(11.4)	0.118
Wound Infection	17 [15]	24(30.3)	0.010
Wound Dehiscence	2(1.7)	6(7.6)	0.052
Compartment Syndrome	10(8.7)	3(3.7)	0.072

SC: Skin-closure only; BBC: Bogota bag closure

A multivariate regression analysis was conducted to assess the association between closure technique (skin-only closure vs. Bogota bag closure) and the likelihood of achieving primary fascial closure (PFC) while controlling for relevant covariates. The results of the multivariate regression analysis revealed that skin-only closure was significantly associated with a higher likelihood of achieving primary fascial closure compared to Bogota bag closure (adjusted odds ratio [OR] = 1.7, 95% confidence interval [CI]: 1.3–3.8, *p* < 0.05). This indicates that, after adjusting for potential confounding variables, patients who underwent skin-only closure were 1.7 times more likely to achieve primary fascial closure than those who underwent Bogota bag closure.

## Discussion

The goal of our study was to assess outcomes after damage control surgery (DCS) based on the initial temporary abdominal closure (TAC), with primary fascial closure (PFC) during the index hospitalization as the key outcome. Skin-only closure (SC) and Bogota bag closure (BBC) are commonly used techniques for obtaining temporary fascial closure in abdominal trauma, minimizing the risk of complications [11]. An overall fascial closure rate of 82% was related to skin-only TAC closure, and the risks of enterocutaneous fistula (6%) and ACS (18.5%) were moderate. Because these data were retrospective and wide-ranging, and many of the studies didn’t cover significant outcomes, caution must be used when interpreting them. Comparing temporary abdominal closure (TAC) with alternative approaches to skin-only closure, patients who underwent skin-only closure showed improved primary fascial closure rates and lower mortality [6]. 

We found a consistent trend of higher male prevalence in both the SC and BBC groups, which aligns with findings from previous studies. Abdominal trauma is more frequently observed in male patients compared to females. The demographic characteristics of both groups in our study were comparable to these previous findings. For instance, Hu et al. conducted a retrospective analysis on trauma patients undergoing damage control surgery and reported a similar pattern with a higher male prevalence of 82.0% and a lower female prevalence of 18.0% [6]. Similarly, a Pakistani study conducted by Muhammad Y. et al. also reported a higher prevalence of males (67.27%) and a lower prevalence of females (32.73%) in cases of open abdominal wounds managed by Bogota bag closure [12]. This observation further supports the notion that abdominal trauma is more frequently observed in male patients compared to females.

Historically, patients treated with SC experienced higher rates of abdominal compartment syndrome and worse outcomes, leading to the abandonment of primary skin closure [13, 14]. However, in our current cohort, the use of the SC technique significantly increased the likelihood of PFC, indicating a twofold improvement in outcomes. On the other hand, patients managed initially with BBC demonstrated significantly worse outcomes despite similar levels of days to closure and a lower incidence of abdominal compartment syndrome compared to patients managed initially with SC. The BBC technique hampers the drainage of intra-abdominal fluid and worsens the lateral retractions of fascial and skin margins, resulting in a lower incidence of primary fascial closure. Our study confirmed these findings, with decreased rates of primary fascial closure using the BBC technique compared to the SC technique [15]. 

A meta-analysis reported that only 34–74% of patients with OA can achieve primary fascial closure, leaving the remaining patients subject to an incisional hernia [16]. In a retrospective review comparing 239 trauma patients receiving damage control surgery, patients who underwent TAC with skin-only closure were contrasted with those who underwent TAC with a Bogota bag, the ABTheraTM VAC system, and Barker’s vacuum packing. Individuals who underwent skin-only closure experienced lower mortality and higher primary fascial closure rates than those who underwent TAC using the other procedures [6]. Sánchez-Lozada et al. conducted a retrospective observational study in which they found that primary closure rates with the Bogota bag ranged from 12 to 82% [17]. Manterola et al. observed that with the TAC with the Bogota bag, the primary fascial closure rate was 39%, the in-hospital mortality rate was 12%, and intra-abdominal sepsis was the most common reason for the contained laparotomy (60%) in their prospective series of 86 patients [18]. 

Patients treated with SC experienced a notable reduction in injury burden, as evidenced by fewer instances of multiple surgeries and reduced reliance on ventilator support. However, it’s important to note that the decision to perform DCS and the choice of TAC at our institution are based on the clinical judgment of the operating surgeon, and there is considerable variation in the frequency and indications for DCS, similar to global trends [19]. Preoperative shock and anemia were substantially less common in patients using this approach, which may have influenced the choice and possibly led to selection bias.

### Limitations

While our study offers valuable insights, several limitations must be acknowledged. Firstly, being a single-center retrospective study, the generalizability of our findings to other healthcare settings may be limited. Secondly, the sample size might lack the statistical power to detect smaller yet clinically meaningful differences between the SC and BBC groups. Thirdly, our exclusion criteria may have introduced selection bias and restricted the generalizability of the findings. Although our reanalysis found no significant association between the number of surgeries and primary fascial closure rates, it’s essential to recognize the potential influence of this factor on our findings. Multiple surgeries could theoretically impact fascial integrity and affect the likelihood of successful primary closure. However, the lack of statistical significance suggests that other factors may have played a more prominent role.

Combining trauma and acute abdomen patients acknowledges the limitations of our study design, based on similar indications for open abdomen management and limited sample sizes within each subgroup. Though this approach may restrict direct comparisons between groups due to the individualized nature of surgical decision-making, our primary aim was to describe outcomes associated with SC and BBC techniques rather than comparing outcomes between trauma and acute abdomen patients. Additionally, our study solely considered data from the index hospitalization, potentially overlooking long-term outcomes and complications.

The retrospective nature and reliance on patient records limit the inclusion of additional severity scores such as SOFA and APACHE. The absence of recorded data on the time lapse between symptom onset or trauma and the first damage control surgery hinders our ability to fully assess their impact on outcomes. Another significant limitation is the potential inaccuracy of preoperative anemia data in trauma cases due to acute variations from hemorrhage, complicating the assessment of anemia status. Lastly, non-randomized patient assignment to closure groups introduced potential confounding variables, impacting the validity of the results.

## Conclusion

In conclusion, our study described the outcomes of skin-only closure (SC) versus Bogota bag closure (BBC) as temporary abdominal closure (TAC) techniques in damage control surgery (DCS). Our findings indicate that SC may offer better outcomes in specific circumstances, as evidenced by significantly higher rates of primary fascial closure (PFC) compared to BBC. However, it’s important to note that the choice between SC and BBC should be carefully considered based on individual patient characteristics and clinical context. Future research should focus on prospective, multicenter studies with larger sample sizes to validate our findings and provide more nuanced evidence on the comparative effectiveness of different TAC techniques. Additionally, exploring novel TAC strategies and assessing their impact on complications, quality of life, and cost-effectiveness will be crucial in advancing the field and informing clinical practice in the management of abdominal trauma.

## Data Availability

The datasets used and/or analyzed during the current study are available from the corresponding author on reasonable request.

## Declarations

### Clinical trial number

N/A.

### Registration of research studies

N/A.
